# Supplementary material for: Metabolic shifts toward glutamine regulate tumor growth, invasion and bioenergetics in ovarian cancer
Source: Mol Syst Biol. 2014 May 5;10(5):728. doi: 10.1002/msb.20134892 (PMC4188042; doi:10.1002/msb.20134892)
Supplement: Supplementary file 1 — Supplementary Figure S1 [file MSB-10-5-728-s07.pdf]

Figure S1

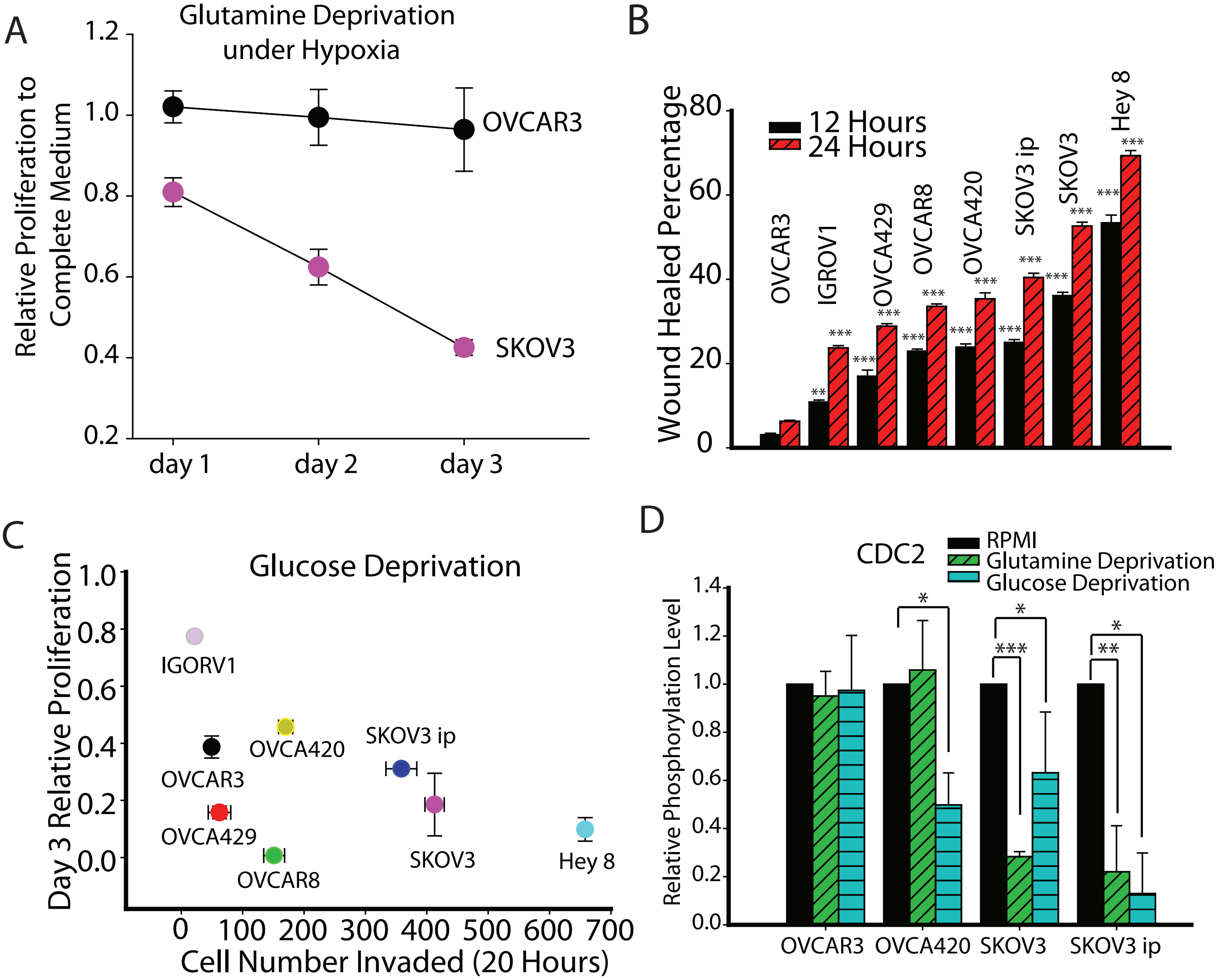

Supplementary Figure S1. (A) Gln deprivation effect on proliferation rate of OVCAR3 and SKOV3 cells under hypoxia (1% O_2) for 24, 48, and 72 hours. (B) Migration ability of a panel of OVCA cells determined using wound healing assay at 12, 24 hours. (C) Relationship between proliferation rate under glucose deprivation and ovarian cancer cells' invasive capacity. (D) CDC2 phosphorylation level in different medium conditions for OVCAR3, OVCA420, SKOV3 and SKOV3ip cells. Data in A-D are expressed as mean ± SD, n≥6, *P<0.05 **P<0.01, ***P<0.001. In B, 1-way ANOVA with Tukey test was used to compare between cell lines, using OVCAR3 as the control.
