# Supplementary material for: Metabolic shifts toward glutamine regulate tumor growth, invasion and bioenergetics in ovarian cancer
Source: Mol Syst Biol. 2014 May 5;10(5):728. doi: 10.1002/msb.20134892 (PMC4188042; doi:10.1002/msb.20134892)
Supplement: Supplementary file 2 — Supplementary Figure S2 [file MSB-10-5-728-s04.pdf]

Fig. S2

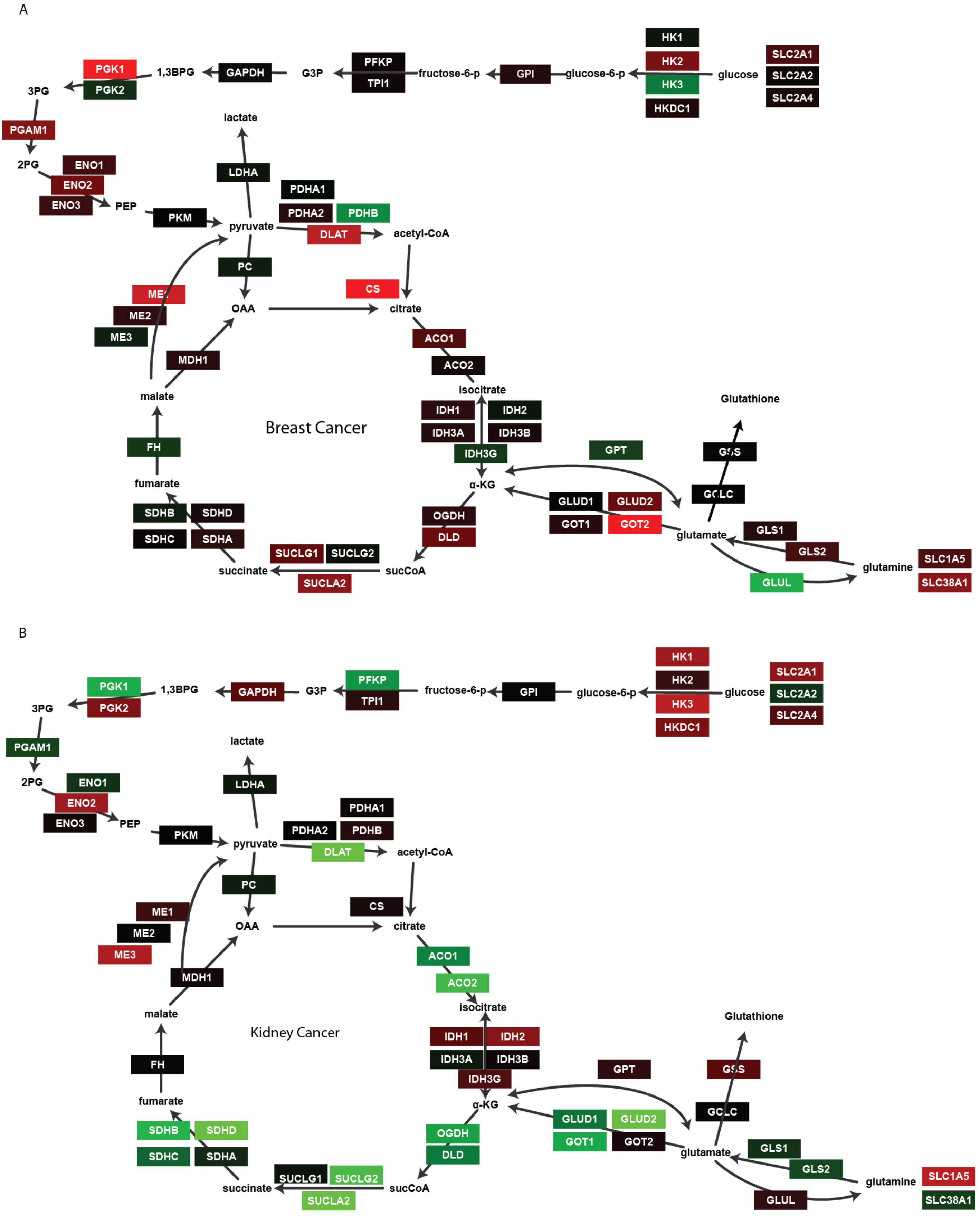

Supplementary Figure S2. Genes in the glutaminolysis and TCA cycle metabolic pathways are associated with higher risk in breast cancer patients (A) and kidney cancer patients (B).
