# Supplementary material for: Metabolic shifts toward glutamine regulate tumor growth, invasion and bioenergetics in ovarian cancer
Source: Mol Syst Biol. 2014 May 5;10(5):728. doi: 10.1002/msb.20134892 (PMC4188042; doi:10.1002/msb.20134892)
Supplement: Supplementary file 3 — Supplementary Figure S3 [file MSB-10-5-728-s10.pdf]

Figure S3

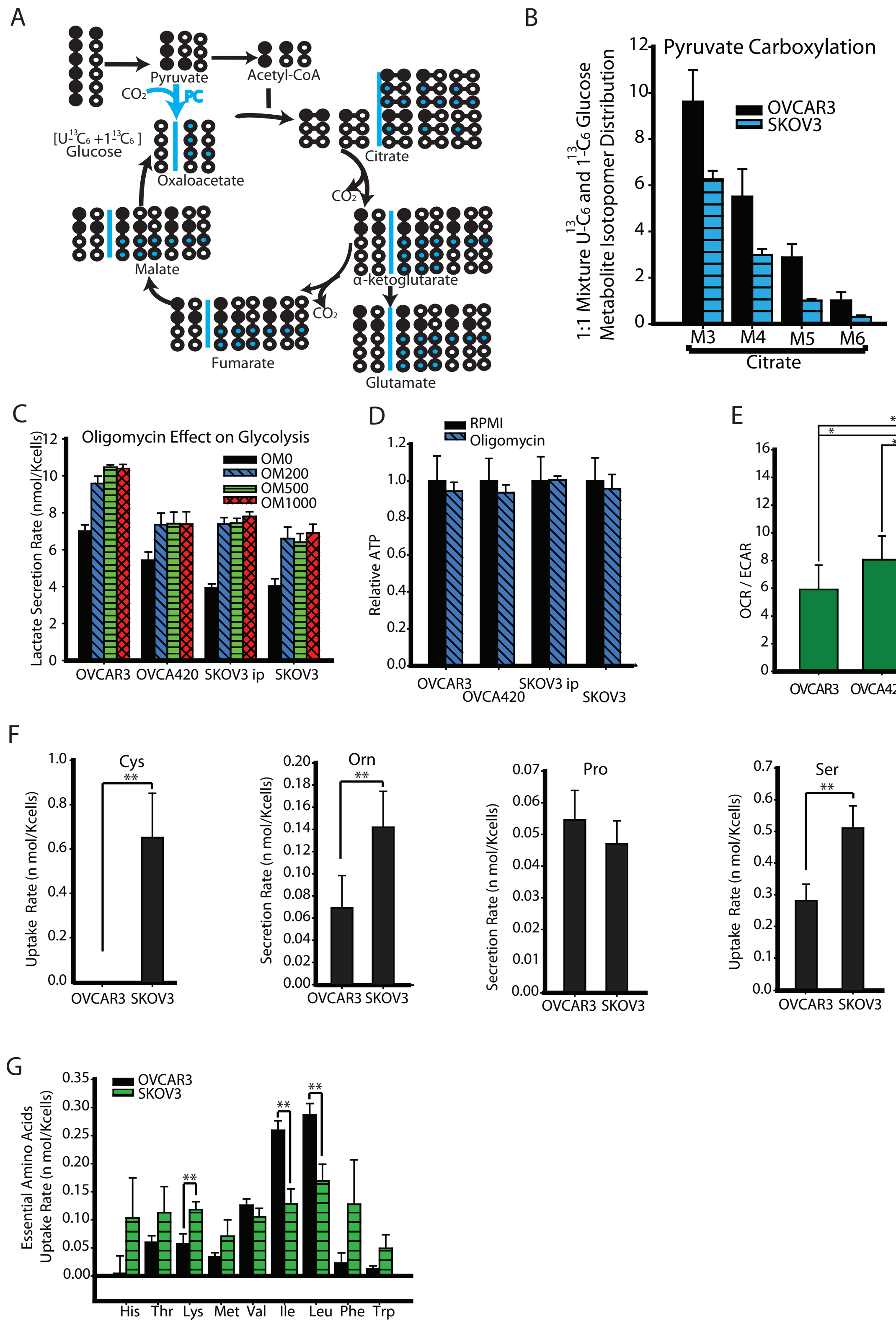

Supplementary Figure S3. (A) Schematic of carbon atom transitions undergoing pyruvate carboxylation using 1:1 mixture of $^{13}C_6$ glucose and 1-^{13}C labeled glucose. Blue circle represents labeled carbon through PC. M3 and M1 pyruvate is directly converted into oxaloacetate by combining with CO_2 through pyruvate carboxylase. (B) Citrate isotopomer distribution using 1:1 mixture of $^{13}C_6$ glucose and 1-^{13}C labeled glucose medium. (C) Effect of varying oligomycin (OM) concentrations (200, 500, 1000 ng/ml) on glycolysis. (D) Oligomycin's effect on ATP content. (E) Basal OCR/ECAR of OVCAR3, OVCA420, SKOV3ip and SKOV3 cells. (F) Uptake/secretion rate of cysteine (Cys), ornithine (Orn), proline (Pro) and serine (Ser) of OVCAR3 and SKOV3 cells in complete medium. (G) Uptake/secretion rate of essential amino acids for OVCAR3 and SKOV3 in complete medium. His, histidine; Thr, threonine; Lys, lysine; Met, methionine; Val, valine; Ile, isoleucine; Leu, leucine; Phe, phenylalanine; Trp, tryptophan Data in B-G are expressed as mean ± SD, n≥9, *P<0.05, **P<0.01, ***P<0.001.
