# Supplementary material for: Metabolic shifts toward glutamine regulate tumor growth, invasion and bioenergetics in ovarian cancer
Source: Mol Syst Biol. 2014 May 5;10(5):728. doi: 10.1002/msb.20134892 (PMC4188042; doi:10.1002/msb.20134892)
Supplement: Supplementary file 4 — Supplementary Figure S4 [file MSB-10-5-728-s02.pdf]

Figure S4

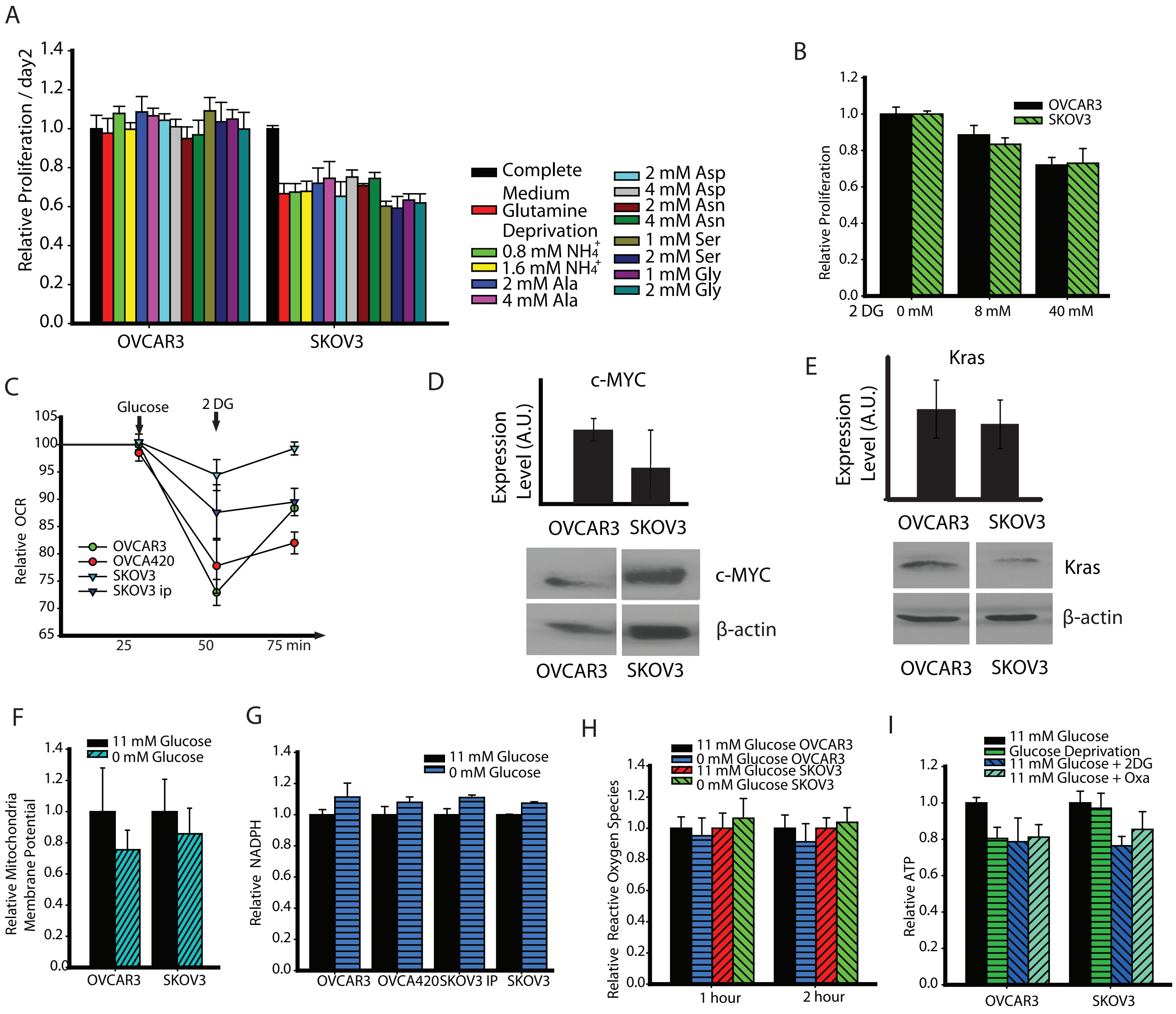

Supplementary Figure S4. (A) Addition of nitrogenous sources (NH4+) and amino acids increasing tumor growth (Ala, Asp, Asn, Ser, Gly) could not rescue Gln deprivation in short term / 48 hours. (B) Effect of different 2-DG concentrations on OVCAR3 and SKOV3's proliferation. (C) Effect of glucose on OCR. Data in A-C are expressed as mean ± SD, n≥9. (D,E) Western blot of c-MYC (D) and k-Ras (E) expression levels in low-invasive OVCA (OVCAR3) and high-invasive (SKOV3) OVCA cells. n≥3. (F) Glucose's effect on mitochondrial membrane potential. (G,H) Glucose's effect on NADPH and ROS generation. (I) Glucose deprivation's effect on ATP content.
