# Supplementary material for: Metabolic shifts toward glutamine regulate tumor growth, invasion and bioenergetics in ovarian cancer
Source: Mol Syst Biol. 2014 May 5;10(5):728. doi: 10.1002/msb.20134892 (PMC4188042; doi:10.1002/msb.20134892)
Supplement: Supplementary file 5 — Supplementary Figure S5 [file MSB-10-5-728-s09.pdf]

Figure S5

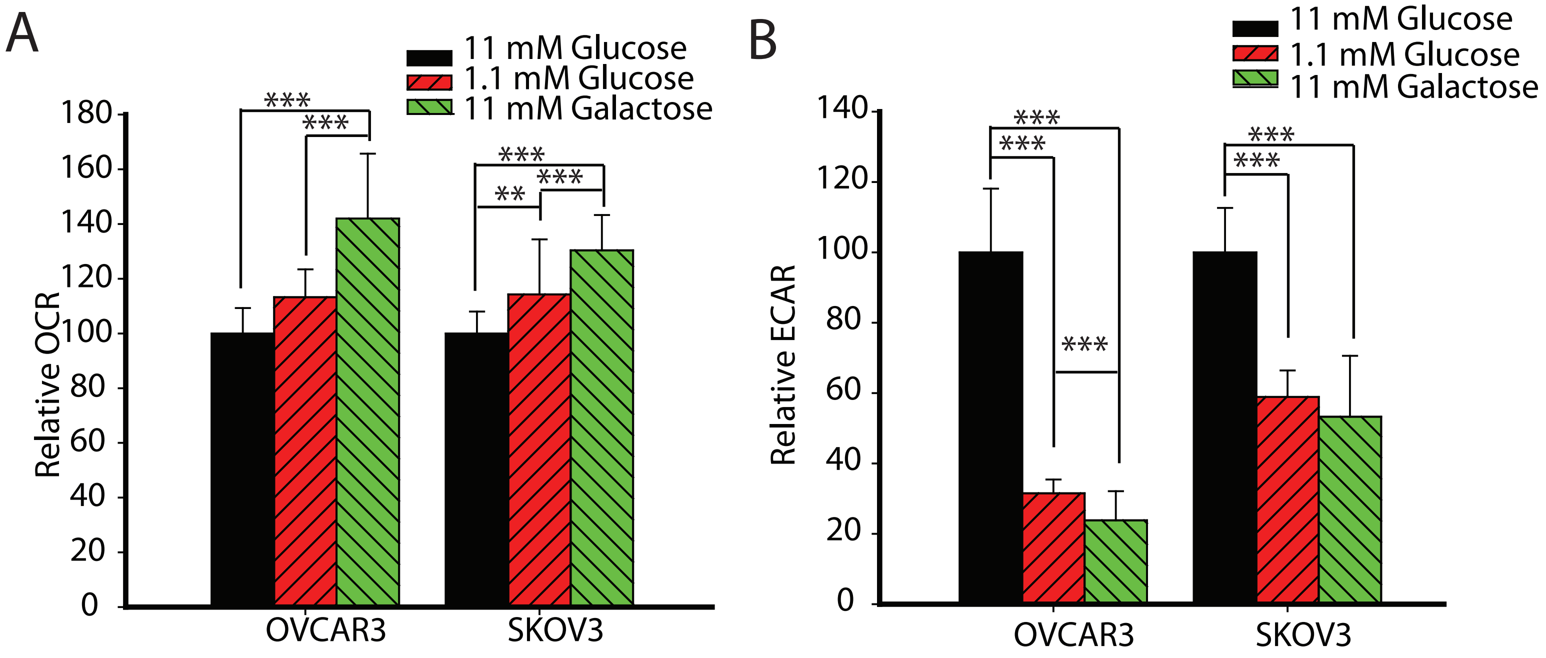

Supplementary Figure S5. (A,B) Galactose and low glucose's effect on OCR (A) and ECAR (B) values. Data in A-B are expressed as mean ± SEM, n≥9, *P<0.05, **P<0.01, ***P<0.001.
