# Supplementary material for: Metabolic shifts toward glutamine regulate tumor growth, invasion and bioenergetics in ovarian cancer
Source: Mol Syst Biol. 2014 May 5;10(5):728. doi: 10.1002/msb.20134892 (PMC4188042; doi:10.1002/msb.20134892)
Supplement: Supplementary file 6 — Supplementary Figure S6 [file MSB-10-5-728-s05.pdf]

Figure S6

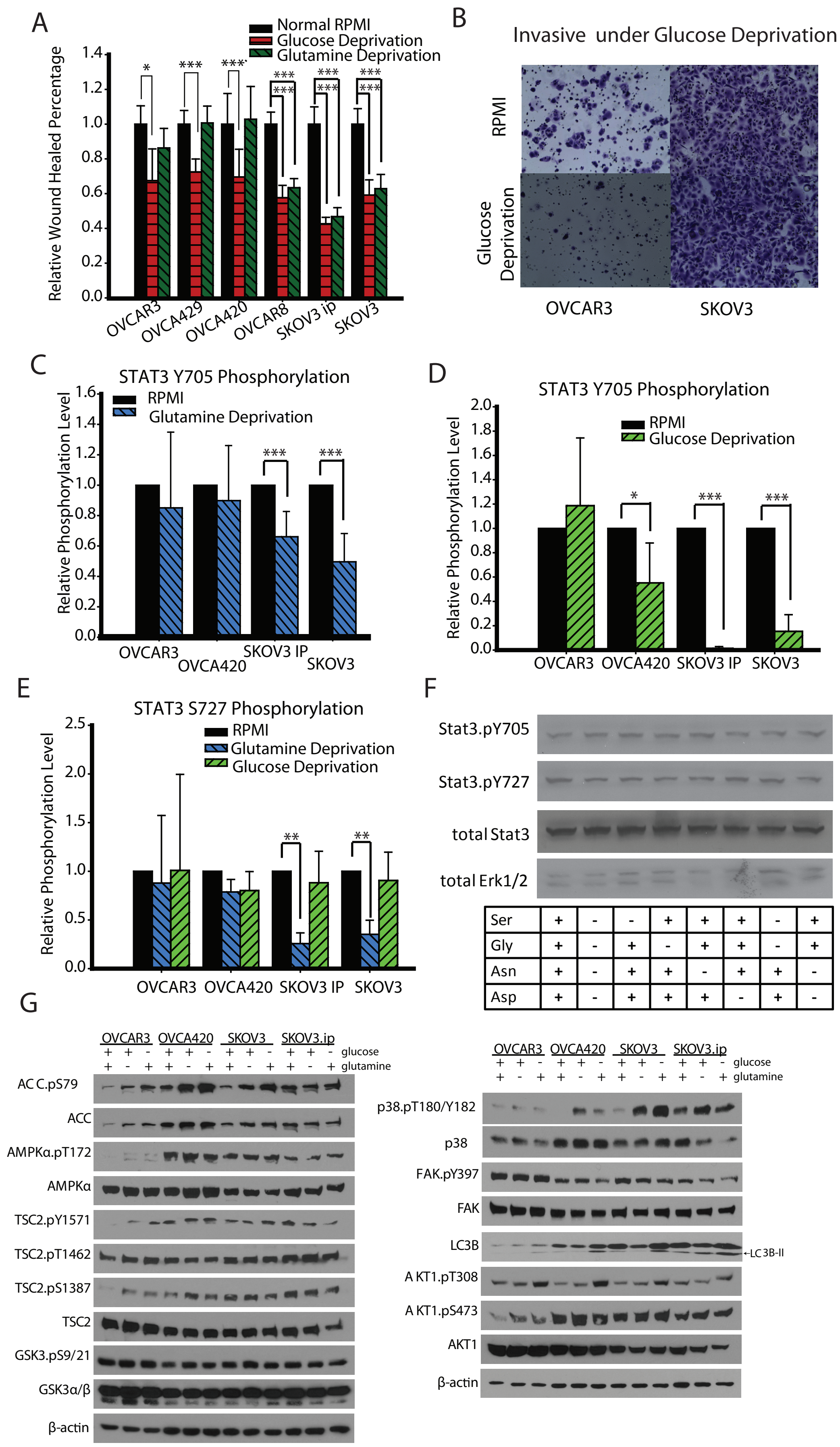

Supplementary Figure S6. (A) Glucose/Gln deprivation's effect on cancer cells' migratory ability. (B) Glucose deprivation's effect on OVCAR3 and SKOV3's invasive capacity. (C-E) STAT3 Y705 phosphorylation level in glutamine / glucose deprivation conditions. STAT3 S727 phosphorylation level in glutamine / glucose deprivation medium conditions. (F) The effect of serine, glycine, asparagine, and aspartate deprivations on STAT3 phosphorylation. Cell culture medium is MEM and was adjusted to similar nutrients component as RPMI. And different amino acids are deprived.(G) Glucose/glutamine deprivation effect on protein expression and phosphorylation level for OVCAR3, OVCA420, SKOV3, SKOV3 ip.
