# Supplementary material for: Metabolic shifts toward glutamine regulate tumor growth, invasion and bioenergetics in ovarian cancer
Source: Mol Syst Biol. 2014 May 5;10(5):728. doi: 10.1002/msb.20134892 (PMC4188042; doi:10.1002/msb.20134892)
Supplement: Supplementary file 7 — Supplementary Figure S7 [file MSB-10-5-728-s06.pdf]

Figure S7

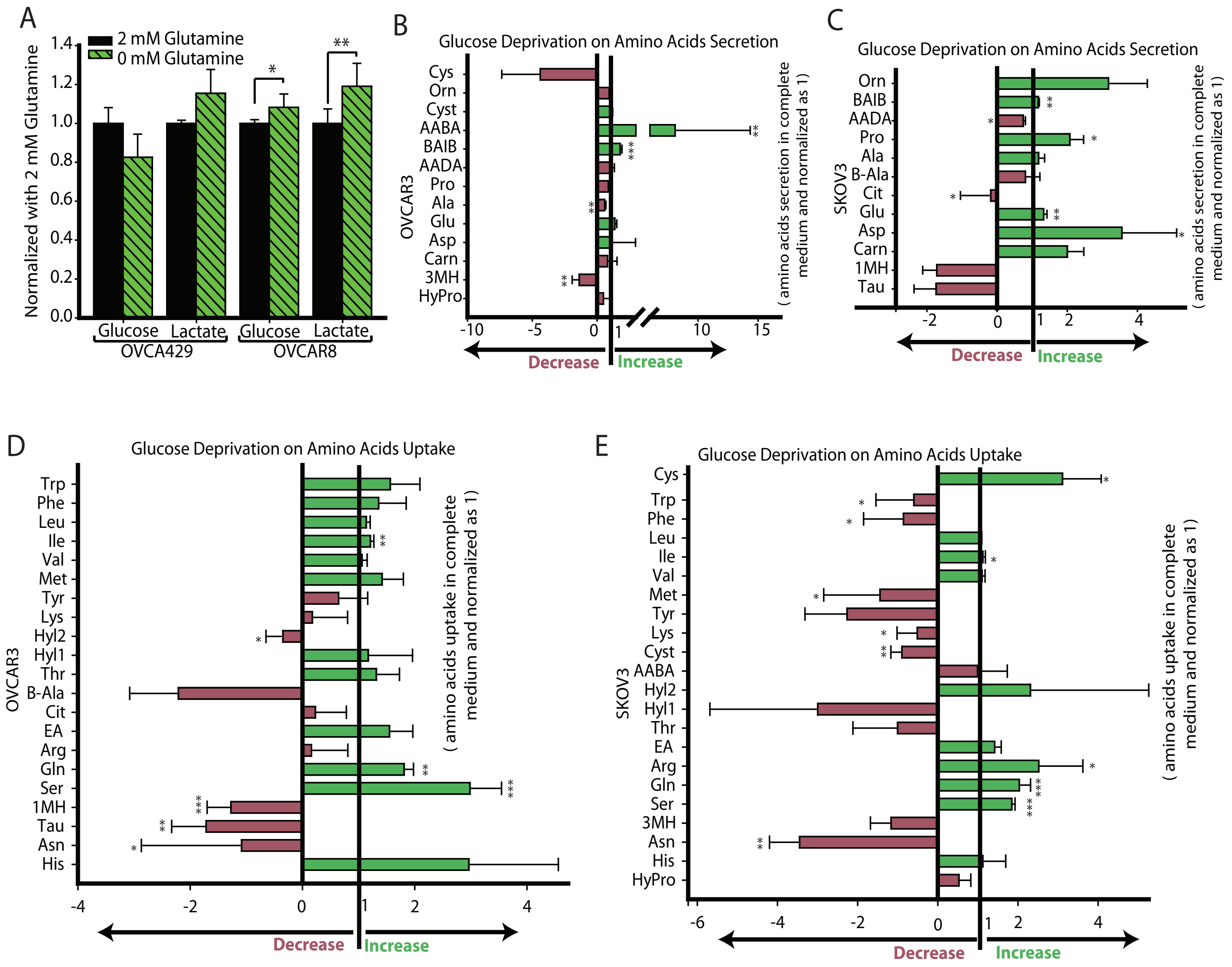

Supplementary Figure S7. (A) Gln's effect on glycolysis for OVCA429 and OVCAR8 cells. (B-E) Amino acid uptake/secretion rate after glucose deprivation measured using UPLC in OVCAR3 (B,D) and SKOV3 (C, E) cells. Data in A-E are expressed as mean ± SEM, n≥6.
