# Supplementary material for: Metabolic shifts toward glutamine regulate tumor growth, invasion and bioenergetics in ovarian cancer
Source: Mol Syst Biol. 2014 May 5;10(5):728. doi: 10.1002/msb.20134892 (PMC4188042; doi:10.1002/msb.20134892)
Supplement: Supplementary file 8 — Supplementary Figure S8 [file MSB-10-5-728-s03.pdf]

Figure S8

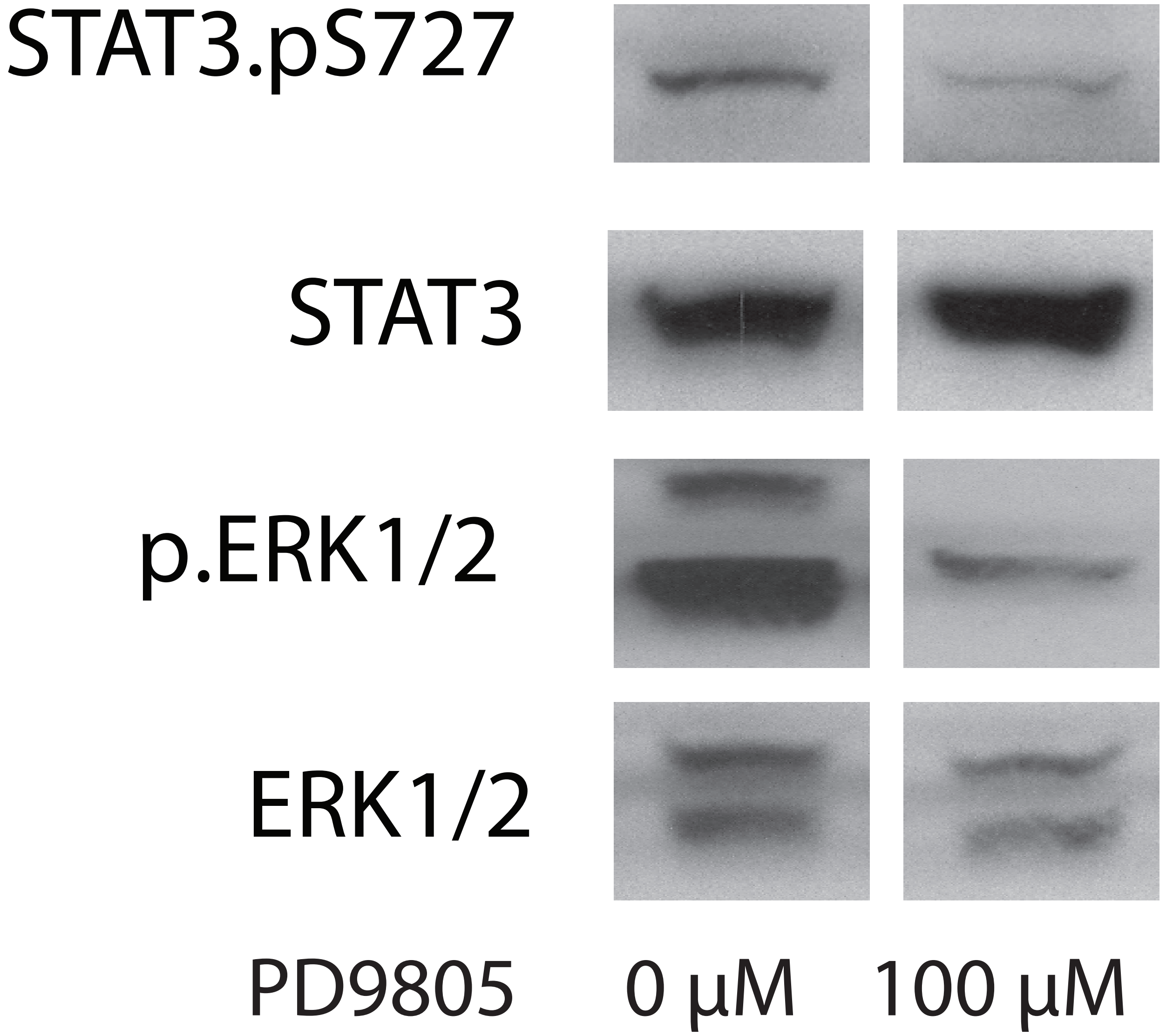

Supplementary Figure S8. STAT3 serine phosphorylation level at the addition of PD98059, a MEK/ERK pathway inhibitor. Here the ERK 1/2 is the loading control.

!
