# Supplementary material for: Metabolic shifts toward glutamine regulate tumor growth, invasion and bioenergetics in ovarian cancer
Source: Mol Syst Biol. 2014 May 5;10(5):728. doi: 10.1002/msb.20134892 (PMC4188042; doi:10.1002/msb.20134892)
Supplement: Supplementary file 9 — Supplementary Table S1 [file MSB-10-5-728-s01.pdf]

Supplementary Table S1: OVCAR3 and SKOV3 Amino Acids Uptake/Secretion Rate in Complete Medium

	OVCAR3 (nmol/Kcells)		SKOV3 (nmol/Kcells)		
	Average	SEM	Average	SEM	P Value
HyPro	-0.2153	0.0771	0.1805	0.2756	
Asn	0.0373	0.0693	0.3816	0.1850	
3MH	-0.0635	0.0889	0.0548	0.1209	
Tau	0.0151	0.0726	-0.0838	0.0883	
1MH	0.0599	0.0564	-0.0338	0.0737	
Carn	-0.0047	0.0019	-0.0150	0.0088	
Arg	0.2538	0.2082	1.0561	0.5308	
EA	0.0036	0.0018	0.0102	0.0021	*
B-Ala	0.0018	0.0015	-0.0039	0.0020	**
AADA	-0.0062	0.0010	-0.0194	0.0018	***
BAIB	-0.0098	0.0032	-0.0608	0.0115	***
Hyl1	0.0006	0.0003	0.0007	0.0006	
Hyl2	0.0003	0.0005	0.0102	0.0051	*
AABA	-0.0005	0.0004	0.0013	0.0004	**
Cyst	-0.0048	0.0014	0.0005	0.0008	***
Tyr	0.0015	0.0174	0.1077	0.0686	

+ : Uptake; - : Secrete

Supplementary Table S1: OVCAR3 and SKOV3's amino acids metabolic fluxes, mean ± SEM, n≥12. *P<0.05, **P<0.01, ***P<0.001. HyPro, hydroxyproline; Asn, Asparagine; 3MH, 3-methylhistidine; Tau, taurine; 1MH, 1-methylhistidine; Arg, arginine; EA, ethanolamine; B-ala, β-alanine; GABA, γ-amino-N-butyric acid; AADA, α-aminoadipic acid; BAIB, β-aminoisobutyric acid; Hyl1, hydroxylysine 1; Hyl2, hydroxylysine 2; AABA, α-amino-N-butyric acid; Cyst, Cystathionine. Tyr, tyrosine.
