# Supplementary figures and images for: Metabolic shifts toward glutamine regulate tumor growth, invasion and bioenergetics in ovarian cancer
Source: Mol Syst Biol. 2014 May 5;10(5):728. doi: 10.1002/msb.20134892 (PMC4188042; doi:10.1002/msb.20134892)

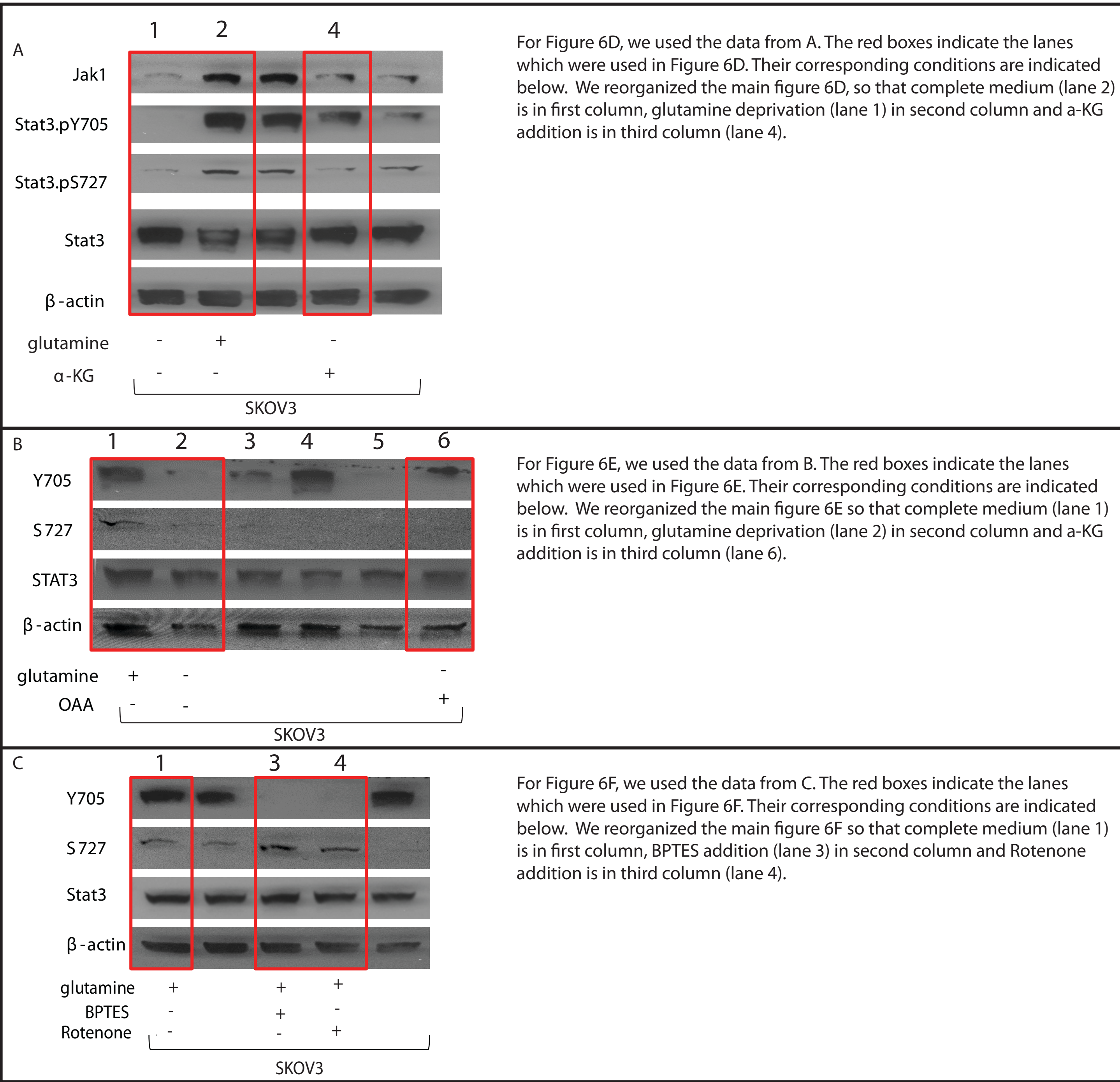

Supplement: Supplementary file 11 — Source Data for Figure 6 D E F [file MSB-10-5-728-s08.pdf]
